# Prebiotic properties and antioxidant effect of crude extracts and polysaccharides from *Agaricus bisporus* and *Pleurotus ostreatus* mushrooms

**DOI:** 10.1038/s41598-025-16152-9

**Published:** 2025-08-24

**Authors:** Yousra A. El-Maradny, Amr S. Abouakkada, Aleya Abdel Gawad Abbass, Amani Farouk Abaza, Esmail M. El-Fakharany

**Affiliations:** 1 Genetic Engineering and Biotechnology Research Institute, City of Scientific Research and Technological Applications (SRTA-City), Alexandria, 21934 Egypt; 2 Microbiology Department, High Institute of Public Health, Alexandria University, Alexandria, Egypt; 3 Protein Research Department, Genetic Engineering and Biotechnology Research Institute, City of Scientific Research and Technological Applications (SRTA-City), Alexandria, 21934 Egypt

**Keywords:** Microbiome, Probiotics, Prebiotics, Carbohydrates, Functional food, Antimicrobials, Bacteriology, Microbiology, Fungi

## Abstract

Modifying the gut microbiome, also known as bacteriotherapy, is a key strategy that uses probiotics, prebiotics, or synbiotics to reduce inflammation and fight infection and colonization by pathogenic bacteria. Various food sources, particularly those rich in *Lactobacillus* species, are well-recognized for their probiotic properties. Edible mushrooms are rich with their nutrient-dense composition, including carbohydrates, proteins, fibers, minerals, vitamins, and lipids, which stand out as a promising bio-source for several biological uses. In this study, four probiotic strains were isolated and identified from food samples: *Lactobacillus acidophilus (L. acidophilus)*, *L. pentosus*, *L. plantarum*, and *L. paracasei*. Then the prebiotic and antioxidant properties of crude and polysaccharide extracts were assessed from two edible mushrooms, *Agaricus bisporus* (brown) and *Pleurotus ostreatus* (oyster). Using the phenol-sulfuric acid method, the ethanol extract of *P. ostreatus* exhibited the highest yields of total carbohydrates and reducing sugars (6.14 and 3.15 mg/mL, respectively). Among the mushroom extracts, the polysaccharide from *A. bisporus* demonstrated the strongest radical scavenging activity (93.73%), with a half-maximal effective concentration (EC_50_) of 0.19 mg/mL, measured using the 1,1-diphenyl-2-picrylhydrazyl (DPPH) radical scavenging method. The prebiotic properties of the mushroom extracts were evaluated by their ability to promote probiotic growth and inhibit pathogenic bacteria. The polysaccharide extracts from *A. bisporus* and *P. ostreatus* significantly stimulated the growth of *L. paracasei* (1.99 and 2.04 nm, respectively). Additionally, the cell-free supernatant from *L. acidophilus* cultured with the *A. bisporus* polysaccharide extract exhibited the highest antimicrobial activity, producing a 36.33 mm inhibition zone against the pathogen *L. monocytogenes*. These findings demonstrate that polysaccharides from *A. bisporus and P. ostreatus* are promising candidates for functional food development. These extracts offer a multifaceted approach to promoting gut health and reducing oxidative stress through selectively stimulating beneficial Lactobacillus species while inhibiting the growth of pathogens and exerting significant antioxidant effects.

## Introduction

**Fig. 1:**
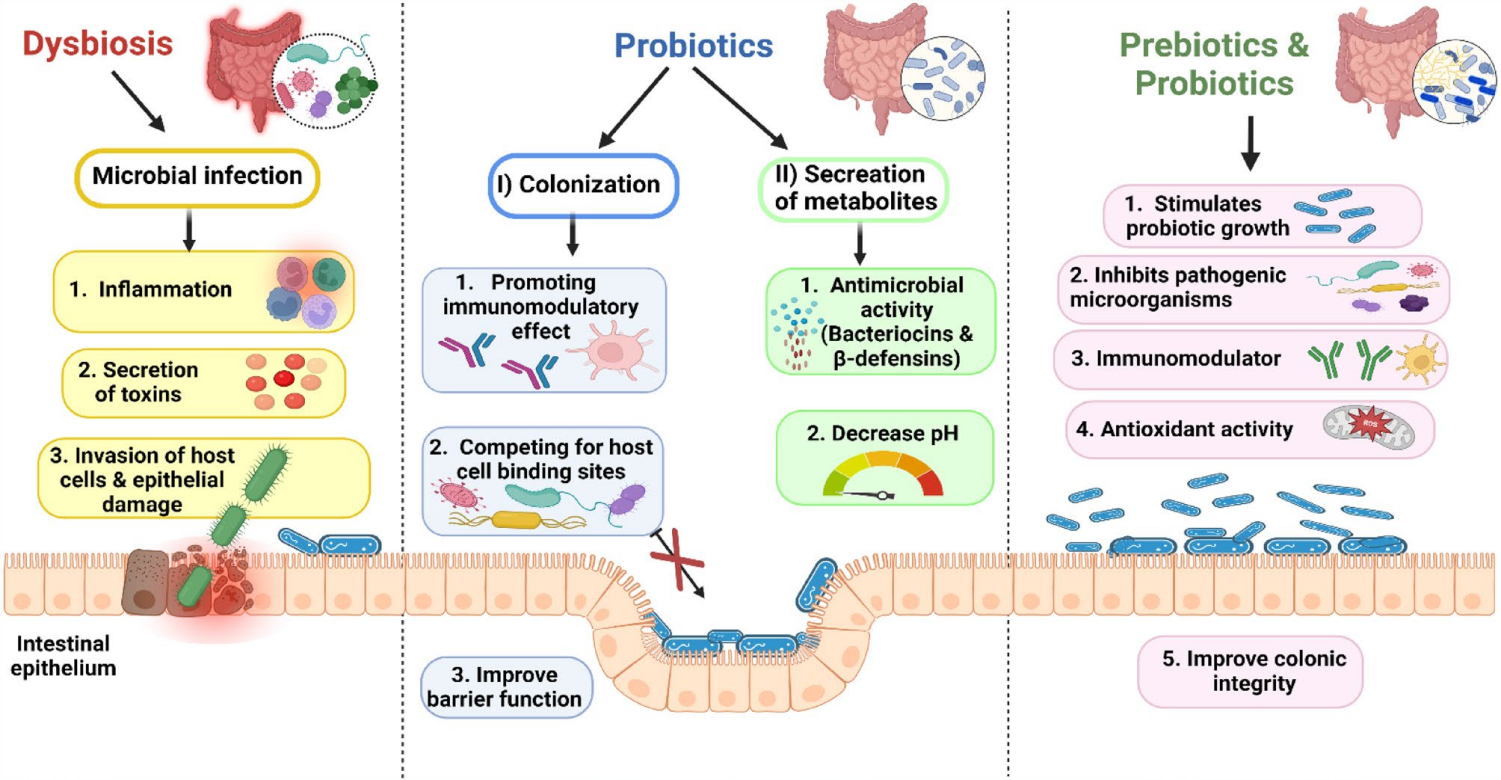
The effect of pathogenic microbial infection and the antimicrobial mechanisms of action of probiotics and prebiotics on intestinal epithelium.

In recent years, the human gut microbiota has been the topic of intensive investigation, with a growing understanding of the different species and their functions^1^. Various variables, such as probiotics (indicating bacteria that boost the microbiome), prebiotics (food compositions rich in oligosaccharides or polysaccharides), and synbiotics (a combination of probiotics and prebiotics), can activate the microbiota in favor of host health. The study of probiotics is critical for understanding the health advantages of gut microbiota^2^. Bacteriotherapy, or modification of the gut microbiome with probiotics, prebiotics, or symbiotics, has a considerable influence on immunomodulation, inflammation reduction, as well as infection and colonization with harmful bacteria^3^. Probiotics are living organisms that, when consumed in the right doses, can improve the health of their hosts. The antipathogenic mechanisms of action of probiotic bacteria include immune system activation and regulation, antagonistic action against pathogenic bacteria, temporary gut colonization, and metabolite production^4^. The *Lactobacillus* genus has received the most attention among the lactic acid bacteria (LAB) for the purpose of choosing probiotic species and strains^5^. Polysaccharides resist decomposition and digestion in saliva, gastric, and small intestinal conditions, presenting a challenge for absorption by the body. This is attributed to the limited number of polysaccharide digestive enzymes (17) encoded by the human genome, with the additional enzymes necessary for polysaccharide digestion being encoded by microbes and their genomes in the human gut^6,7^. Dysbiosis is the result of an unbalanced gut microbiota, which results in disease and microbial infection. Prebiotics are indigestible dietary ingredients (such as mushrooms) that can act directly as antioxidants or immunomodulators or indirectly by selectively stimulating the gut microbiota to grow and thus benefiting their hosts^8^. Prebiotics are mostly composed of oligosaccharides and fiber. According to a new trend in food science and technology, prebiotics have been linked to modulating the human gut microbiota and reducing the risk of diabetes, obesity, and tumors^9^. The effects of pathogenic microbes during dysbiosis, probiotics, and prebiotics on human intestinal epithelial cells were presented in Fig. 1. Mushrooms are rich in polysaccharides like chitin and β-glucans, as well as galactans, making them an excellent source of prebiotics^10,11^. Mushrooms serve as prebiotics, promoting the formation of the gut microbiota and so providing health advantages to the host. Mushrooms have a long tradition of use in many countries. They are foods full of amino acids, different minerals, enzymes, and physiologically active polysaccharides. Mushrooms have over a hundred different therapeutic properties. Recently, there has been a significant increase in interest in the antioxidant properties of mushrooms and their polysaccharides^12^. Mushrooms have been reported as useful in preventing diseases such as hypertension, hypercholesterolemia, diabetes, microbial infections, inflammations, allergies, cancer, and have hepatoprotective properties^13–16^. The nutritional value of *Pleurotus* spp. has been well known; according to recent studies, the genus is low in fat, rich in fiber, and has nutritional levels of carbohydrates, amino acids, and minerals that are significantly higher than those of other resources^17^. *Ganoderma lucidium* (GL) is a well-known macrofungal that is shown to lower obesity in rats via altering gut microbiota composition^18^. By considerably increasing the numbers of lactic acid-producing bacteria and improving the intestine’s health, the use of *Agaricus bisporus* mushrooms modifies the composition, performance, and morphology of the intestinal microbiota, as well as antioxidant levels in turkey poults^18^. While the general benefits of edible mushrooms are known, critical gaps in understanding still exist. Specifically, few studies have directly compared the bioactivity of crude versus purified polysaccharide extracts from common edible mushrooms like *A. bisporus* and *P. ostreatus*. Furthermore, the strain-specific synergistic effects between these prebiotics and different *Lactobacillus* species against key human pathogens remain underexplored. Therefore, this study was designed to systematically evaluate and compare the prebiotic, antipathogenic, and antioxidant properties of crude versus polysaccharide extracts from *A. bisporus* and *P. ostreatus*. By elucidating these specific bioactivities, this study aims to advance the understanding of how these common edible mushrooms can be utilized as functional food components to modulate the gut microbiome, inhibit enteric pathogens, and mitigate oxidative stress.

## Materials and methods

### Experimental design overview

The study was conducted in a multi-step process. First, potential probiotic *Lactobacillus* strains were isolated from 100 different food samples and subsequently identified to the species level using Matrix-Assisted Laser Desorption/Ionization Time-of-Flight (MALDI-TOF) mass spectrometry. Four distinct isolates were selected for further analysis. In parallel, crude and polysaccharide extracts were prepared from *A. bisporus* and *P. ostreatus* mushrooms. These extracts were then biochemically characterized by quantifying their total carbohydrate and reducing sugar content, and their antioxidant capacity was assessed using DPPH and ABTS assays. To evaluate prebiotic potential, the mushroom extracts were supplemented into the growth media of the previously identified *Lactobacillus* strains. Probiotic growth stimulation was determined by measuring the change in optical density. Finally, the cell-free supernatants from the *Lactobacillus* cultures that had been supplemented with the mushroom extracts were collected and tested for their ability to inhibit the growth of various pathogenic bacteria^19,20^.

### Chemicals, media, and instruments

All used chemicals were analytical grade from Sigma-Aldrich, St. Louis, MO, USA and Fluka (Darmstadt, Germany). The media used in this study were commercially available as dehydrated media from Oxoid, UK and Himedia, India. Instruments used in this study included Spectrophotometry INNO-M (LTEK Co., Ltd Instruments, Sunil Technopia 903-ho, 555, Republic of Korea 13215), Incubator (Heraeus, D-63450, Germany), Centrifuge (Eppendorf 5810 R, Germany), Rotary evaporator (Yamato BM 100, Japan), Homogenizer (T 25 digital ULTRA-TURRAX^®^ IKA, labortechnik, Staufen, Germany), lyophilizer (TELSTAR, ORTODOS-50, Spain), MALDI-TOF-MS (Bruker daltonik MALDI biotyper, Germany), and Stomacher (Seward, UK).

### Isolation of lactobacilli strains

A total of 100 food samples of the most consumed types of yoghurt, cheese, buttermilk, pickles, and honey were randomly purchased from various markets in Alexandria, Egypt. All the collected samples were transported within two hours in an ice box to the laboratory for processing. Twenty-five g of each purchased food sample were mixed with 225 mL of buffered peptone water (BPW) in a sterile stomacher bag and homogenized using a stomacher (Seward, UK). This mixture was considered a 10^−1^ dilution. Then, tenfold serial dilutions of 10^−2^, 10^−3^, and 10^−4^ were performed using 9 mL Man, Rogosa and Sharpe (MRS) broth tubes. One mL from each homogenized food sample and each dilution (10^−2^, 10^−3^, and 10^−4^) was streaked onto the surface of an MRS agar plate. The plates were incubated at 37 °C for 48 h under anaerobic conditions using an anaerogen gas pack and anaerobic jar. After incubation, individual colonies were selected and transferred into sterile MRS broth tubes. The selected colonies were purified by streaking onto the surface of a sterile MRS plate using a sterile bacteriological loop. The isolates were then examined according to their colony morphology, catalase reaction, and Gram stain^21^. Isolates that appeared as round, opaque, creamy, or milky white colonies on the surface of MRS agar plates and then, when viewed under the microscope after Gram staining, appeared as gram positive rods and were catalase negative were identified using MALDI-TOF biotype. Then, the stocks of identified LAB were kept at −80◦C in cryovials of MRS broth containing 20% (v/v) glycerol.

### Microorganisms

Four strains of prebiotic lactobacilli (*L. acidophilus*, *L. pentosus*, *L. plantarum*, and *L. paracasei*) and five pathogenic bacteria (*Staphylococcus aureus* (ATCC 25923), *Escherichia coli* (ATCC 25922), *Shigella dysenteriae*,* Listeria monocytogenes* (ATCC 13932) and methicillin-resistant *Staphylococcus aureus* (MRSA)) were cultivated in an appropriate medium, MRS medium, Nutrient agar (NA) or Nutrient broth (NB).

### Extraction of mushroom extract and its polysaccharides

Two kinds of edible mushrooms were purchased from the Egyptian local market, the brown *A. bisporus* and *P. ostreatus*. For crude extract, 500 g of fresh mushroom fruiting bodies were washed, homogenized, and extracted with phosphate-buffered saline (PBS), pH 7.4, overnight at 4 °C^22^. The homogenate was centrifuged at 4000 rpm for 30 min, and the resulting supernatant was collected and filtered. About 500 g of edible mushrooms were washed and dried at 105 °C overnight for polysaccharide extraction, then completely mixed for 5 min in a blender. Each sample was divided into 10 g and placed in separate test tubes. All materials were extracted using 30 mL of a 1:4 v/v combination of distilled water and ethanol 95%. The mushrooms were shaken for 4 h at 80 °C and 150 rotations per minute. Then, it was centrifuged at 4000 rpm at 25 ± 2 °C for 15 min, and all supernatants were then maintained at 4 °C for total carbohydrate and total reducing sugar analyses^20^. To obtain the dried fractions, all mushroom constituents were lyophilized and stored at 4 °C until required.

### Total carbohydrates and reducing sugar determination

The phenol-sulfuric acid technique was used to determine the carbohydrate concentration of the crude and ethanolic mushroom extracts^23^. In this experiment, a mixture of 0.25 mL of mushroom sample, 1.25 mL of concentrated sulfuric acid, and 0.25 mL of 5% phenol was heated to 100 °C for 5 min, then cooled at 25 ± 2 °C. The total carbohydrate contents were measured at an absorbance of 490 nm, and a standard and blank sample were analyzed using glucose and distilled water, respectively, instead of mushroom samples.

All mushroom extracts were determined for total reducing sugar using a 3,5-dinitrosalicylic acid (DNS) assay according to^24^. After the extraction process, each extracted mushroom sample was centrifuged at 4000 rpm at 25 ± 2 °C for 15 min. A supernatant aliquot of one mL was mixed with one mL of DNS reagent and incubated at 100 °C for 5 min. After cooling the mixture to 25 ± 2 °C, the reducing sugar was measured using spectrophotometry at 540 nm absorbance. The standard and blank samples were prepared and analyzed in the same way, except for adding one mL of glucose to the standard sample and one mL of distilled water to the blank sample.

## Antioxidant activity

Mushroom extracts were investigated for their antioxidant capacity in two different assays, namely, the ABTS^+^ and 1,1-diphenyl-2-picrylhydrazyl (DPPH) radical scavenging activities.

## a. Scavenging activity on 1,1-diphenyl-2-picrylhydrazyl (DPPH) radicals

The scavenging activity of the ethanol and water extracts from mushrooms on DPPH radicals was measured according to the method of^25^ with some modifications. An aliquot of 0.5 mL of 0.1 mM DPPH radical (Sigma) in methanol was added to a test tube with one mL of mushroom crude and polysaccharide extracts of different concentrations (1.5 to 9 mg/mL). Methanol or water was used instead of the mushroom sample as a control, and Ascorbic acid was used as a standard. The reaction mixture was vortex mixed at 25 ± 2 °C, and the absorbance (Abs) was determined immediately after mixing by measuring at 520 nm with a spectrophotometer. The scavenging activity percent (SA%) on DPPH radicals was calculated by Eq. (1).

1 $${\rm DPPH \:SA\%= (1- Abs\: in\: the\: presence\: of \:sample/Abs\: in\: the\: absence\: of\: sample) \times100.}$$

### b. ABTS radical scavenging assay

Another method for determining the free radical scavenging activity of mushroom crude and polysaccharide extracts was the ABTS radical cation decolorization assay^26^. ABTS^+^ cation radical was produced by the reaction between 7 mM ABTS^+^ in water and 2.45 mM potassium persulfate (1:1), stored in the dark at 25 ± 2 °C for 12–16 h before use. The ABTS^+^ solution was then diluted with methanol to obtain an absorbance of 0.700 at 734 nm. After the addition of 5 µL of plant extract to 3.995 mL of diluted ABTS^+^ solution, the absorbance was measured 30 min after the initial mixing. An appropriate solvent blank was run in each assay. All the measurements were carried out at least three times. Percent inhibition of absorbance at 734 nm was calculated using the formula, Eq. 2.

2 $${\rm ABTS^{+} scavenging effect (\%) = ((AB-AA)/AB) \times 100}$$

where AB is absorbance of ABTS radical + methanol; AA is absorbance of ABTS radical + sample extract/standard. Ascorbic acid was used as standard substance.

### Prebiotic properties

#### Probiotic growth stimulation

*L. acidophilus*, *L. pentosus*, *L. plantarum*, and *L. paracasei* were cultured at 37 °C for 48 h in MRS broth (used as the control), compared with culture medium supplemented with 2.5 and 5 mg/mL of each mushroom extract and a commercial prebiotic compound such as inulin. After incubation, the cultures were quantified by measuring the optical cell density using spectrophotometry at 620 nm^27^.

#### Inhibition of pathogenic bacteria by probiotics

To evaluate the antipathogenic potential of the synbiotic combinations, a three-step method was employed. First, prebiotic cultures were prepared by growing each *Lactobacillus* strain for 48 h at 37 °C in MRS broth supplemented with 5 mg/mL of each mushroom extract. Second, the cultures were centrifuged (8000 rpm, 4 °C, 15 min), and the cell-free supernatant, containing metabolites produced by the probiotics from fermenting the mushroom extracts, was collected. Third, pathogenic bacteria (*L. monocytogenes*, *E. coli*, *S. dysenteriae*, *S. aureus*, and MRSA) were cultured on Muller-Hinton agar (MHA) plates. Sterile filter paper discs were impregnated with 20 µL of the collected supernatant and placed on the plates. Antimicrobial activity was determined by measuring the diameter of the clear zone of inhibition after 24 h of incubation at 37 °C. After 24 h of incubation at 37 °C, inhibition efficiency was determined by comparing the diameter of the clear zone on the plate containing probiotic supernatant to that of the media lacking probiotics and mushroom extracts (negative control) and the culture containing a commercial prebiotic compound (positive control)^28^.

#### Statistical analysis

All quantitative experiments were performed in triplicate, and results are expressed as mean ± standard deviation (SD). Group comparisons were performed using either an independent samples t-test (for two groups) or a one-way analysis of variance (ANOVA) followed by Tukey’s post-hoc test for multiple comparisons (for more than two groups), using SPSS software (version 16.0). For antioxidant assays, the half-maximal inhibitory concentration (IC_50_) values were determined by fitting the data to a non-linear regression model using GraphPad Prism software (version 8.0). In all analyses, a p-value of less than 0.05 was considered to indicate statistical significance.

## Results

### Distribution of the LAB isolates from food samples

A total of 100 food samples examined, from yoghurt, cheese, buttermilk, pickles, and honey, summarized in Table 1. Pickles yielded the highest percentage of LAB isolates (95.0%). This was followed by yoghurt samples (70.0%), which represented the highest percentage among the tested dairy products. Cheese samples were the least likely to yield LAB isolates (35.0%). No LAB isolates were recovered from any of the examined honey samples. Out of the 58 isolated LABs, *L. pentosus* and *L. plantarum* accounted for 48.3% of all identified LAB isolates. Yoghurt was the only source of *L. paracasei* isolates in this study, with a percentage of 9.5%. Also, *L. pentosus* was isolated only from pickle samples (79.0%). *L. acidophilus* isolates were recovered from yoghurt and buttermilk samples with percentages of 23.8% and 9.1%, respectively, while *L. plantarum* was isolated from cheese and buttermilk with percentages of 71.0% and 72.7%, respectively.

**Table 1:** Distribution of *Lactobacillus* species isolated from various food samples.

Food Source	Samples Tested	Samples with LAB (%)	*L. acidophilus* (%)	*L. pentosus* (%)	*L. plantarum* (%)	*L. paracasei* (%)
Yoghurt	20	70.0	23.8	0	0	9.5
Cheese	20	35.0	0	0	71.0	0
Buttermilk	20	60.0	9.1	0	72.7	0
Pickles	20	95.0	0	79.0	0	0
Honey	20	0.0	0	0	0	0

### Total carbohydrate and total reducing sugar determination

The crude and polysaccharide extracts were used for the determination of carbohydrates and reducing sugar amounts. As shown in Table 2, the polysaccharides extract of oyster mushrooms had the highest carbohydrates and reducing sugars of 6.14 and 3.15 mg/mL, respectively.

**Table 2:** Total polysaccharide and reducing sugar content of mushroom extracts.

Extract Type	Total polysaccharides (mg/mL)	Total reducing sugar (mg/mL)
AB B (Crude)	0.88^d^ ± 0.00049	0.62^d^ ± 0.002
PO (Crude)	2.90^b^ ± 0.02	0.65^c^ ± 0.003
BP (Ethanol Precipitate)	1.01^c^ ± 0.10	0.76^b^ ± 0.001
OP (Ethanol Precipitate)	6.14^a^ ± 0.16	3.15^a^ ± 0.03

All data are expressed as mean ± SD and considered significantly different at *p* < 0.05. Crude *Agaricus bisporus* brown (AB B), crude *Pleurotus ostreatus* (PO), ethanol polysaccharide extracts of *Agaricus bisporus* (BP), and ethanol polysaccharide extracts of *Pleurotus ostreatus* (OP).

### Antioxidant activity

**Fig. 2:**
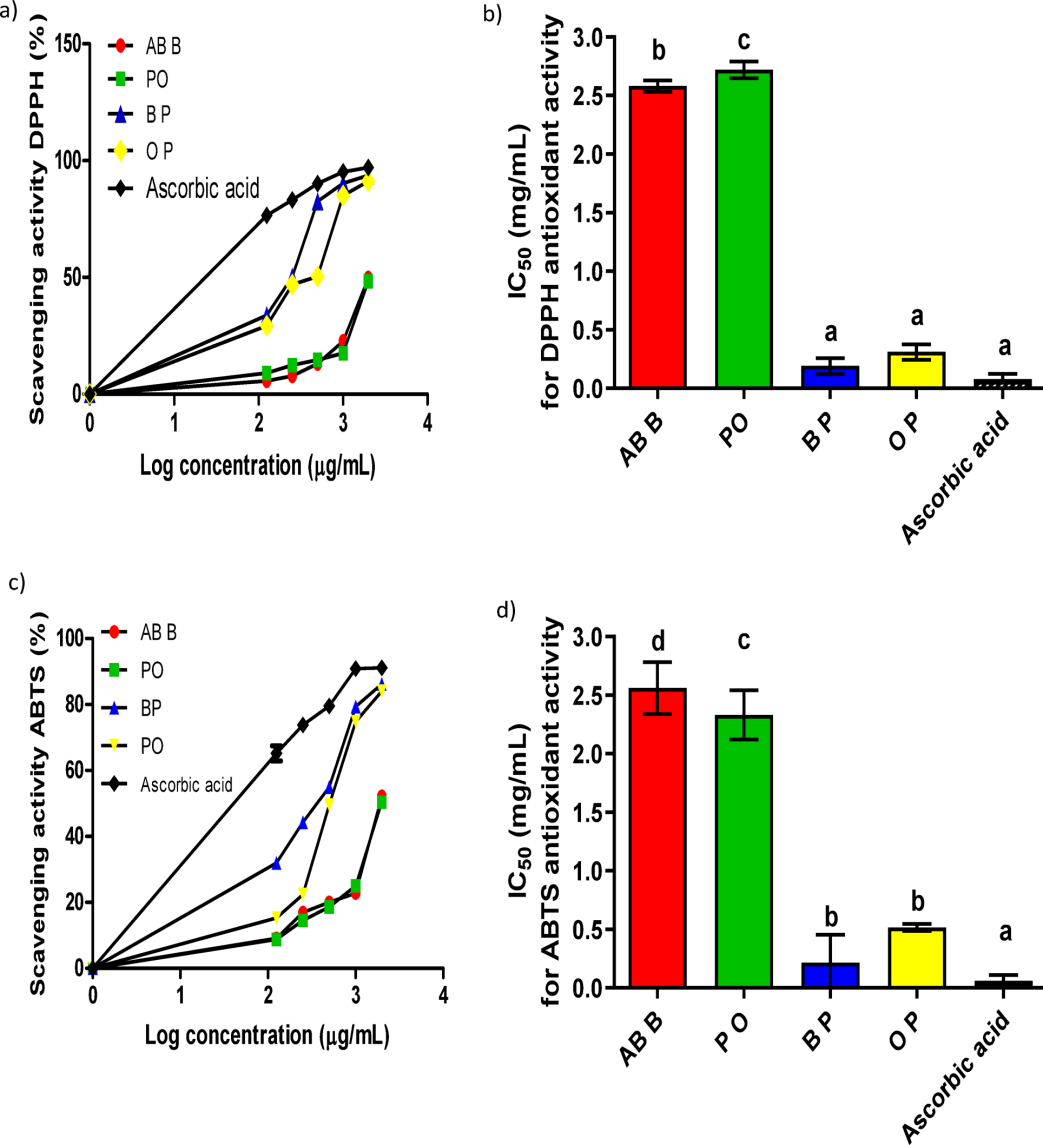
Antioxidant activity **(a)** Dose response scavenging activity of mushroom crude and polysaccharide extracts using DPPH radical assay, **(b)** The IC_50_ for mushroom extracts using DPPH radical assay, **(c)** Dose response scavenging activity of mushroom crude and polysaccharide extracts using ABTS radical assay, **(d)** The IC_50_ for mushroom extracts using ABTS radical assay. All data are expressed as mean ± SD and considered significantly different at *p* < 0.05. Crude *Agaricus bisporus* brown (AB B), crude *Pleurotus ostreatus* (PO), ethanol polysaccharide extracts of *Agaricus bisporus* (BP), and ethanol polysaccharide extracts of *Pleurotus ostreatus* (OP).

Mushrooms are widely recognized for their antioxidant properties. In this study, two assays, DPPH and ABTS radical scavenging tests, were evaluated the antioxidative potential of crude and polysaccharide extracts from mushrooms in comparison to ascorbic acid. The results, illustrated in Fig. 2a and c, indicate that the scavenging activity of these extracts is concentration-dependent. Notably, the polysaccharide extracts of both brown and oyster mushrooms exhibited significantly higher antioxidant activity than the crude extracts in the DPPH assay, with EC_50_ values of 0.19 mg/mL and 0.31 mg/mL, respectively, as shown in Fig. 2b. Similarly, when assessed using the ABTS assay, the polysaccharide extracts displayed IC_50_ values of 0.315 mg/mL and 0.516 mg/mL, respectively, as depicted in Fig. 2d.

### Probiotic growth stimulation

**Fig. 3:**
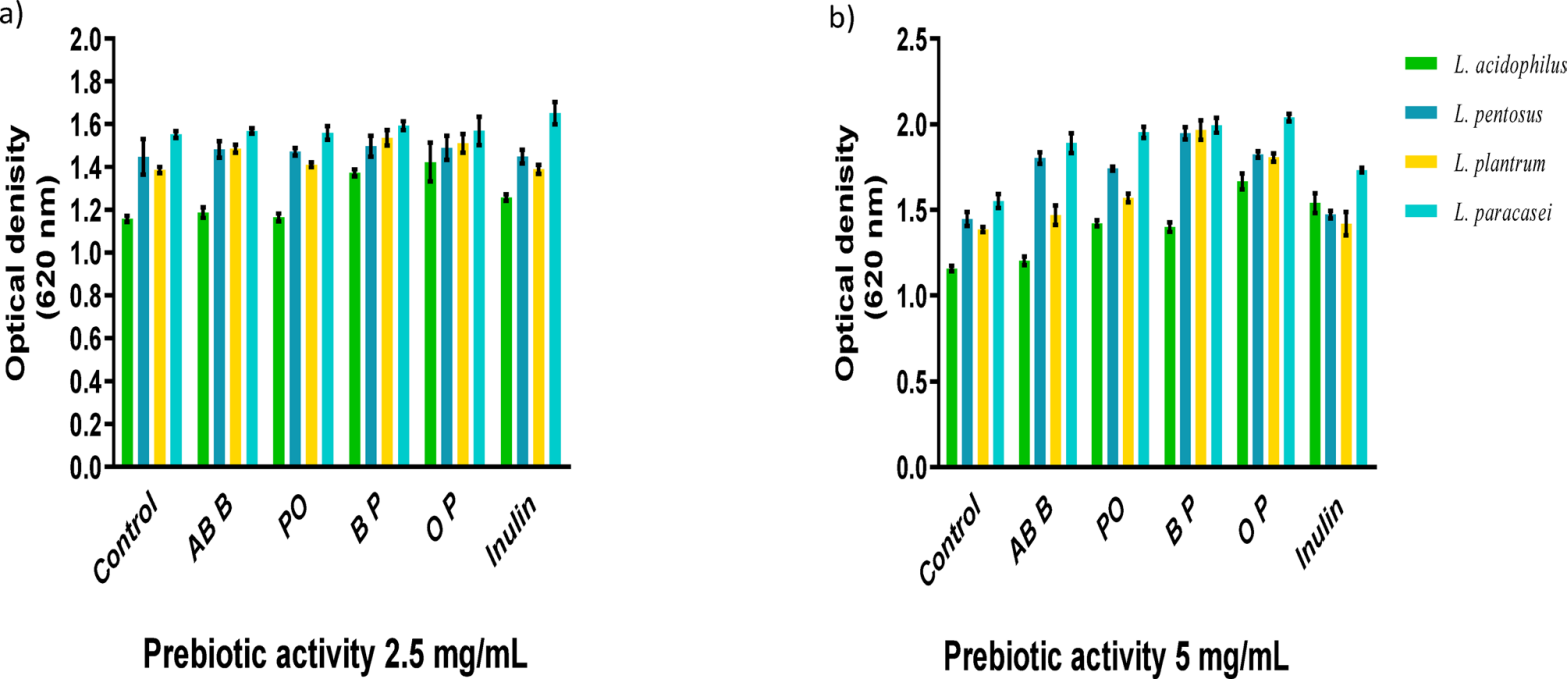
Prebiotic growth stimulation, **(a)** Using 2.5 mg/mL of mushroom extracts, **(b)** Using 5 mg/mL of mushroom extracts. All data are expressed as mean ± SD and considered significantly different at *p* < 0.05. Crude *Agaricus bisporus* brown (AB B), crude *Pleurotus ostreatus* (PO), ethanol polysaccharide extracts of *Agaricus bisporus* (BP), and ethanol polysaccharide extracts of *Pleurotus ostreatus* (OP).

Following the cultivation of lactobacilli with and without mushroom crude and polysaccharide extracts, the results were compared to inulin as a commercial prebiotic standard. Notably, cultivation with 2.5 mg/mL of polysaccharide extracts from *Agaricus bisporus* and *Pleurotus ostreatus* led to the highest growth stimulation of *L. paracasei*, with values of 1.59 and 1.57, respectively, as shown in Fig. 3a. Furthermore, the addition of 5 mg/mL of polysaccharide extracts significantly enhanced the prebiotic activity across all studied lactobacilli strains, as illustrated in Fig. 3b. Among these, the polysaccharide extracts from brown and oyster mushrooms showed the highest stimulation of *L. paracasei* growth, achieving values of 1.99 and 2.04, respectively.

### Inhibition of pathogenic bacteria by probiotics

**Fig. 4:**
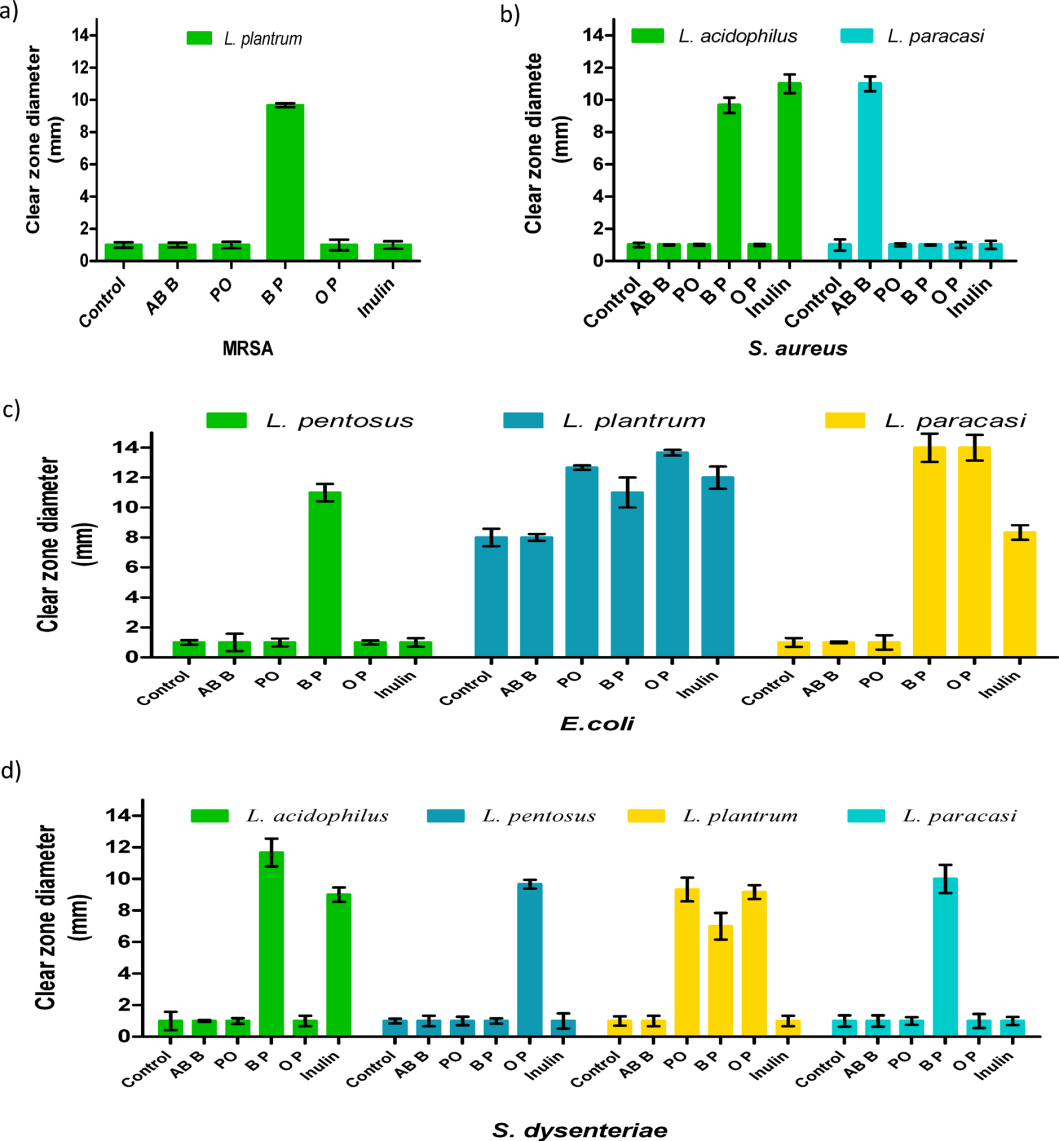

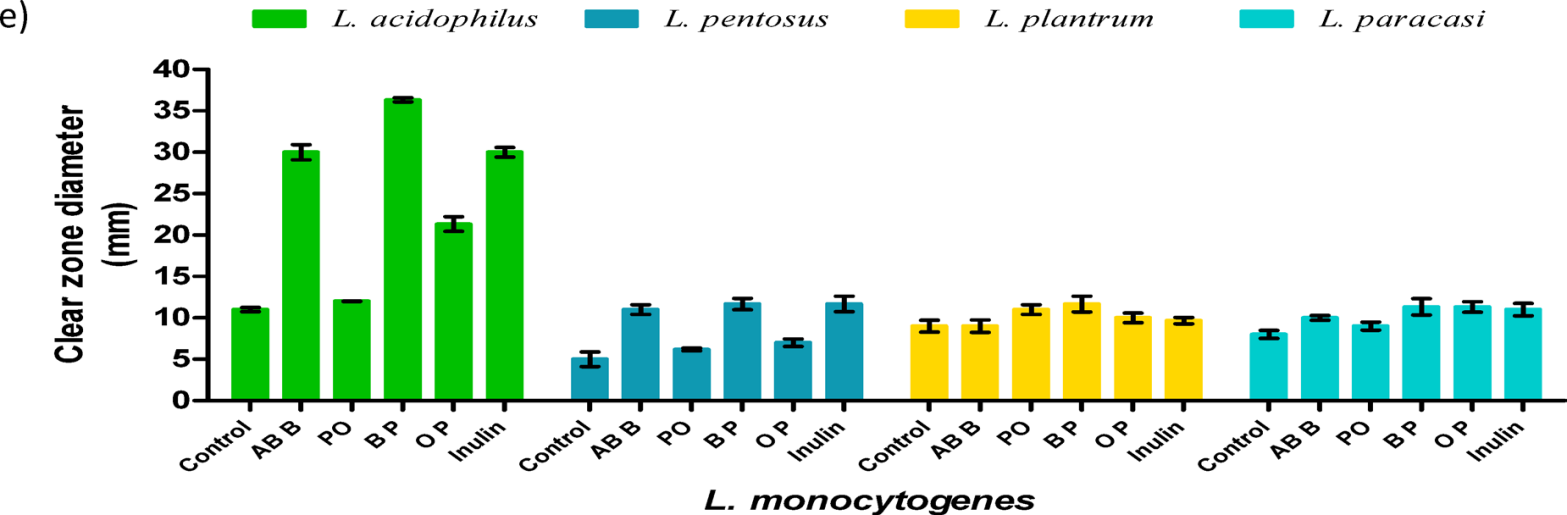
**Clear zone width of pathogenic growth inhibition; (a)** MRSA, **(b)** *S. aureus*, **(c)** *E. coli*, **(d)** *S. dysenteriae*, **(e)** *L. monocytogenes*. The inhibition was observed using supernatants from *Lactobacillus* species cultured in MRS media fortified with mushroom crude and polysaccharide extracts. The control group consists of *Lactobacillus* supernatant without fortification. All data are expressed as mean ± SD. Crude *Agaricus bisporus* brown (AB B), crude *Pleurotus ostreatus* (PO), ethanol polysaccharide extracts of *Agaricus bisporus* (BP), and ethanol polysaccharide extracts of *Pleurotus ostreatus* (OP).

The inhibition of pathogenic growth by prebiotic cultivations is presented in Fig. 4**(a-e)**. The culture media of LAB containing mushroom crude extracts, polysaccharides, or inulin demonstrated varying levels of antipathogenic effects. Among these, *L. acidophilus* cultured with mushroom extracts produced the largest clear zones of *L. monocytogenes* inhibition when grown with the crude extract and polysaccharides of *A. bisporus* and the polysaccharide of *P. ostreatus*, measuring 30.00, 36.33, and 21.33 mm, respectively, as shown in Fig. 4e. Following this, *L. paracasei* treated with polysaccharide extracts of *A. bisporus* and *P. ostreatus* against *E. coli* resulted in clear zones with diameters of 14.00 mm, as depicted in Fig. 4c.

## Discussion

This study demonstrates that polysaccharide extracts from *A. bisporus* and *P. ostreatus* possess significant antioxidant, prebiotic, and antipathogenic properties, making them promising candidates for nutraceutical development. A key finding was that purification substantially enhanced these activities, with purified extracts consistently outperforming their crude counterparts.

The high carbohydrate and reducing sugar yields from *P. ostreatus* align with previous reports^20^. In the antioxidant assays, the purified extracts showed potent radical scavenging capacity. The IC_50_ value for the *A. bisporus* extract against the DPPH radical was 0.19 mg/mL. This activity is comparable to the 0.38 mg/mL reported by Liu et al.^29^although it indicates lower potency than the 81.3 µg/mL reported by Elmastas et al.^30^. In contrast, the *P. ostreatus* extract exhibited a potent IC_50_ of 0.31 mg/mL, which is nearly seven times more effective than the 2.14 mg/mL reported by Yan et al.^12^. Such variations in antioxidant capacity likely arise from differences in the specific mushroom strains, growing conditions, or extraction methodologies used across studies. Nevertheless, the strong ability of these extracts to neutralize free radicals supports their potential application as functional ingredients to mitigate oxidative stress, a key factor in inflammation and aging^17,31^.

The prebiotic potential of the mushroom extracts was confirmed by their ability to stimulate the growth of *Lactobacillus* strains, particularly at higher concentrations. This finding is consistent with previous research demonstrating that mushroom-derived polysaccharides can be more effective prebiotics than conventional fibers like inulin^3,20^. The enhanced production of short-chain fatty acids (SCFAs) by the polysaccharide-fed lactobacilli further underscores this prebiotic activity. SCFAs are critical for maintaining gut homeostasis, supporting immune function, and inhibiting pathogens by lowering intestinal pH^31,32^. Therefore, these polysaccharides represent a promising tool to enhance digestive health and support colonic integrity, although in vivo studies are needed to confirm their impact on gut microbiota composition and metabolic activity^3,18,33^.

The potent antibacterial effect observed in this study can be attributed to a synergy between the LAB and mushroom polysaccharides. The antimicrobial effects of LAB are largely attributed to environmental acidification and the secretion of bacteriocins^34,35^. These findings suggested that the mushroom polysaccharides act as a prebiotic, stimulating *Lactobacillus* growth and thereby increasing the production of these inhibitory substances.

The most significant novel finding of this work is the potent, strain-specific synbiotic effect observed against *L. monocytogenes*. Specifically, the supernatant from *L. acidophilus* fortified with *A. bisporus* polysaccharides demonstrated a powerful inhibitory activity that was not seen with other combinations. This suggests a highly specific interaction, where *A. bisporus* polysaccharides serve as an optimal prebiotic substrate for *L. acidophilus*, leading to the enhanced production of a potent cocktail of antimicrobial metabolites.

This efficacy can be attributed to a “multi-pronged attack” mechanism. Primarily, the fermentation of polysaccharides leads to the secretion of organic acids and bacteriocins, which create a low-pH environment hostile to pathogens^36,37^. Additionally, the production of SCFAs has been independently associated with reduced pathogen virulence and colonization^36–38^. This combined assault overwhelms the defenses of *L. monocytogenes* far more effectively than it does other pathogens. For instance, gram-negative bacteria like *E. coli* and *S. dysenteriae* are protected by an outer membrane, while the bacteriocins produced by *L. acidophilus* may not be the primary target for other gram-positive pathogens like *S. aureus* or MRSA^39–42^.

Our finding that this effect is highly strain-specific is strongly supported by the literature. While the results of this study align with Sawangwan et al.^20^who also reported inhibition of a pathogen (*S. paratyphi*) by *L. acidophilus* cultured with mushroom extracts, the higher efficacy in this study points to the uniqueness of the strain and substrate combination. This aligns perfectly with the conclusions of both Tejero-Sariñena et al.^38^ and Burgos et al.^43^who have demonstrated that the antimicrobial and probiotic potential of LAB is dictated not by the species alone, but by the unique genetic makeup of an individual strain. The work by Burgos et al.^43^for example, showed that *L. curvatus* SC076 was significantly more effective against *L. monocytogenes* than other LAB from the same environment, reinforcing that these highly specific, potent interactions are a key area for discovery in probiotic research.

## Conclusion

This study demonstrates that polysaccharide-rich extracts from *A. bisporus* and *P. ostreatus* are multi-functional bioactive ingredients with significant potential for the functional food industry. These extracts possess potent intrinsic antioxidant properties and, more importantly, act as advanced prebiotics. The primary finding of this work is the clear prebiotic capacity of these extracts, which selectively stimulated the growth of beneficial lactobacilli. The present study goes a crucial step further by revealing the functional consequence of this prebiotic action. This study demonstrated a powerful, indirect antipathogenic effect, where cell-free supernatants from *Lactobacillus* cultures enriched with these polysaccharides were highly effective at inhibiting pathogens, most notably *L. monocytogenes*. This illustrates a sophisticated synbiotic mechanism where the polysaccharides do not simply act as “food” for probiotics, but actively enhance their ability to produce protective metabolites, thereby creating a more defensive gut environment. The *A. bisporus* extract, when combined with *L. acidophilus*, emerged as a particularly superior combination, highlighting the specificity of these probiotic-prebiotic interactions. However, this study has several limitations that open avenues for future research. The experiments were conducted entirely *in- vitro*, which does not fully replicate the complex, anaerobic ecosystem of the human gut. Furthermore, the number of probiotic strains tested was limited, and the antipathogenic assay was qualitative. Future work should therefore focus on *in- vivo* models to assess the impact of these synbiotics on complex gut microbiota. Additionally, quantitative measures, such as minimum inhibitory concentration (MIC), are needed to determine the precise potency of the antimicrobial effect, and a broader range of probiotic strains should be screened to optimize these interactions for gastrointestinal health. Ultimately, positive outcomes from these preclinical studies would be essential to justify and guide the design of future human clinical trials to validate these findings in a practical, therapeutic context.

## Data Availability

The authors confirm that the data underpinning the results of this study are contained within the manuscript. Raw data files are accessible in alternative formats upon request from the corresponding author.

## References

[1] Li, D. et al. Diet-gut microbiota-epigenetics in metabolic diseases: from mechanisms to therapeutics. *Biomed. Pharmacother.* **153**, 113290 (2022). 35724509 10.1016/j.biopha.2022.113290

[2] He, M. & Shi, B. Gut microbiota as a potential target of metabolic syndrome: The role of probiotics and prebiotics. *Cell and Bioscience* vol. 7 Preprint at (2017). 10.1186/s13578-017-0183-1 10.1186/s13578-017-0183-1 PMC5655955 29090088

[3] Nowak, R., Nowacka-Jechalke, N., Juda, M. & Malm, A. The preliminary study of prebiotic potential of Polish wild mushroom polysaccharides: the stimulation effect on Lactobacillus strains growth. *Eur. J. Nutr.* **57**, 1511–1521 (2018). 28353071 10.1007/s00394-017-1436-9 PMC5959981

[4] Monteagudo-Mera, A., Rastall, R. A., Gibson, G. R., Charalampopoulos, D. & Chatzifragkou, A. Adhesion mechanisms mediated by probiotics and prebiotics and their potential impact on human health. *Applied Microbiology and Biotechnology* vol. 103 6463–6472 Preprint at (2019). 10.1007/s00253-019-09978-7 10.1007/s00253-019-09978-7 PMC6667406 31267231

[5] de Albuquerque, T. M. R. et al. In vitro characterization of Lactobacillus strains isolated from fruit processing by-products as potential probiotics. *Probiotics Antimicrob. Proteins*. **10**, 704–716 (2018). 28836171 10.1007/s12602-017-9318-2

[6] Zhao, J. et al. The Interaction between Mushroom Polysaccharides and Gut Microbiota and Their Effect on Human Health: A Review. *Biology* vol. 12 Preprint at (2023). 10.3390/biology12010122 10.3390/biology12010122 PMC9856211 36671814

[7] Fu, C. et al. Simulated gastrointestinal digestion and gut microbiota fermentation of polysaccharides from agaricus bisporus. *Food Chem.* **418**, 135849 (2023). 36963137 10.1016/j.foodchem.2023.135849

[8] Teferra, T. F. Possible actions of inulin as prebiotic polysaccharide: A review. *Food Frontiers* vol. 2 407–416 Preprint at (2021). 10.1002/fft2.92

[9] Hutkins, R. W. et al. Prebiotics: Why definitions matter. *Current Opinion in Biotechnology* vol. 37 1–7 Preprint at (2016). 10.1016/j.copbio.2015.09.001 10.1016/j.copbio.2015.09.001 PMC4744122 26431716

[10] Jayachandran, M., Xiao, J. & Xu, B. A critical review on health promoting benefits of edible mushrooms through gut microbiota. *International Journal of Molecular Sciences* vol. 18 Preprint at (2017). 10.3390/ijms18091934 10.3390/ijms18091934 PMC5618583 28885559

[11] Vetter, J. The Mushroom Glucans: Molecules of High Biological and Medicinal Importance. *Foods* vol. 12 Preprint at (2023). 10.3390/foods12051009 10.3390/foods12051009 PMC10000499 36900525

[12] Yan, J. et al. Analyses of active antioxidant polysaccharides from four edible mushrooms. *Int. J. Biol. Macromol.* **123**, 945–956 (2019). 30447375 10.1016/j.ijbiomac.2018.11.079

[13] Yu, S., Weaver, V., Martin, K. & Cantorna, M. T. The effects of whole mushrooms during inflammation. *BMC Immunol.* **10**, 12 (2009). 19232107 10.1186/1471-2172-10-12 PMC2649035

[14] Finimundy, T. C. et al. Aqueous extracts of lentinula Edodes and pleurotus sajor-caju exhibit high antioxidant capability and promising in vitro antitumor activity. *Nutr. Res.* **33**, 76–84 (2013). 23351413 10.1016/j.nutres.2012.11.005

[15] Saied, E. M. et al. A comprehensive review about the molecular structure of severe acute respiratory syndrome coronavirus 2 (Sars-cov-2): Insights into natural products against covid-19. *Pharmaceutics* vol. 13 Preprint at (2021). 10.3390/pharmaceutics13111759 10.3390/pharmaceutics13111759 PMC8624722 34834174

[16] Liu, Y., Zhou, Y., Liu, M., Wang, Q. & Li, Y. Extraction optimization, characterization, antioxidant and Immunomodulatory activities of a novel polysaccharide from the wild mushroom paxillus involutus. *Int. J. Biol. Macromol.* **112**, 326–332 (2018). 29371151 10.1016/j.ijbiomac.2018.01.132

[17] Rodrigues Barbosa, J., dos Santos Freitas, M. M. & da Silva Martins, L. H. & de Carvalho, R. N. Polysaccharides of mushroom Pleurotus spp.: New extraction techniques, biological activities and development of new technologies. *Carbohydrate Polymers* vol. 229 Preprint at (2020). 10.1016/j.carbpol.2019.115550 10.1016/j.carbpol.2019.115550 31826512

[18] Giannenas, I. et al. Consumption of agaricus bisporus mushroom affects the performance, intestinal microbiota composition and morphology, and antioxidant status of Turkey Poults. *Anim. Feed Sci. Technol.* **165**, 218–229 (2011).

[19] Panya, M., Kaewraemruaen, C., Saenwang, P. & Pimboon, P. Evaluation of Prebiotic Potential of Crude Polysaccharides Extracted from Wild Lentinus polychrous and Lentinus squarrosulus and Their Application for a Formulation of a Novel Lyophilized Synbiotic. *Foods* 13, (2024). 10.3390/foods13020287 PMC10815080 38254588

[20] Sawangwan, T., Wansanit, W., Pattani, L. & Noysang, C. Study of prebiotic properties from edible mushroom extraction. *Agric. Nat. Resour.* **52**, 519–524 (2018).

[21] Gómez, N. C., Ramiro, J. M. P. & Quecan, B. X. V. & De Melo franco, B. D. G. Use of potential probiotic lactic acid bacteria (LAB) biofilms for the control of *Listeria monocytogenes*, *Salmonella* typhimurium, and *Escherichia coli* O157: H7 biofilms formation. *Front. Microbiol.* **7**, 863 (2016). 27375584 10.3389/fmicb.2016.00863 PMC4901071

[22] Blessing Bukola, O. *Ultrasonic-Assisted Extraction and Antibacterial Activites of Protein Recovred from White Button Mushroom (Agaricus Bisporus)*. *International Journal of PharmTech Research CODEN (USA): IJPRIF* vol. 8 (2015).

[23] Dubois, M., Gilles, K. A., Hamilton, J. K., Rebers, P. A. & Smith, F. Colorimetric method for determination of sugars and related substances. *Anal. Chem.* **28**, 350–356 (1956).

[24] Miller, G. L. Use of Dinitrosalicylic acid reagent for determination of reducing sugar. *Anal. Chem.* **31**, 426–428 (1959).

[25] Chu, Y. H., Chang, C. L. & Hsu, H. F. Flavonoid content of several vegetables and their antioxidant activity. *J. Sci. Food Agric.* **80**, 561–566 (2000).

[26] Re, R., Pellegrini, N., Proteggente, A., Pannala, A. & Yang, M. C. Antioxidant activity applying an improved ABTS radical cation decolorization assay. *Free Radic Biol. Med.* **26**, 1231–1237 (1999). 10381194 10.1016/s0891-5849(98)00315-3

[27] Siragusa, S. et al. Taxonomic structure and monitoring of the dominant population of lactic acid bacteria during wheat flour sourdough type I propagation using Lactobacillus sanfranciscensis starters. *Appl. Environ. Microbiol.* **75**, 1099–1109 (2009). 19088320 10.1128/AEM.01524-08 PMC2643576

[28] Rousseau, V., Lepargneur, J. P., Roques, C., Remaud, S. M. & Paul, F. Prebiotic effects of oligosaccharides on selected vaginal lactobacilli and pathogenic microorganisms. *Anaerobe* **11**, 145–153 (2005). 16701545 10.1016/j.anaerobe.2004.12.002

[29] Liu, J., Jia, L., Kan, J. & Jin, C. hai. In vitro and in vivo antioxidant activity of ethanolic extract of white button mushroom (Agaricus bisporus). *Food Chem. Toxicol.* **51**, 310–316 (2013). 23099505 10.1016/j.fct.2012.10.014

[30] Elmastas, M., Isildak, O., Turkekul, I. & Temur, N. Determination of antioxidant activity and antioxidant compounds in wild edible mushrooms. *J. Food Compos. Anal.* **20**, 337–345 (2007).

[31] Steve, N. T. I. & Hui, S. Mushrooms bioactive as prebiotics to modulate gut microbiota in relationships with causes and prevention of liver diseases (Review). *International Journal of Medicinal Mushrooms* vol. 22 509–519 Preprint at (2020). 10.1615/IntJMedMushrooms.2020034706 10.1615/IntJMedMushrooms.2020034706 32865893

[32] Singdevsachan, S. K. et al. Mushroom polysaccharides as potential prebiotics with their antitumor and immunomodulating properties: A review. *Bioactive Carbohydrates and Dietary Fibre* vol. 7 1–14 Preprint at (2016). 10.1016/j.bcdf.2015.11.001

[33] Türsen Uthan, E., Yamaç, M. & Yildiz, Z. In vitro prebiotic activity of polysaccharides extracted from Edible / Medicinal macrofungi species. *J. Fungus Nisan*. **13**, 15–29 (2022).

[34] Bungenstock, L., Abdulmawjood, A. & Reich, F. Evaluation of antibacterial properties of lactic acid bacteria from traditionally and industrially produced fermented sausages from Germany. *PLoS One* **15**, e0230345 (2020). 32160253 10.1371/journal.pone.0230345 PMC7065787

[35] Bamisi, O. E., Ogidi, C. O. & Akinyele, B. J. Antimicrobial metabolites from probiotics, pleurotus ostreatus and their co-cultures against foodborne pathogens isolated from ready-to-eat foods. *Ann. Microbiol.* **74**, 31 (2024).

[36] Ricke, S. C. Perspectives on the use of organic acids and short chain fatty acids as antimicrobials. *Poult. Sci.* **82**, 632–639 (2003). 12710485 10.1093/ps/82.4.632

[37] Zhang, Z., Lv, J., Pan, L. & Zhang, Y. Roles and applications of probiotic Lactobacillus strains. *Applied Microbiology and Biotechnology* vol. 102 8135–8143 Preprint at (2018). 10.1007/s00253-018-9217-9 10.1007/s00253-018-9217-9 30032432

[38] Tejero-Sariñena, S., Barlow, J., Costabile, A., Gibson, G. R. & Rowland, I. Antipathogenic activity of probiotics against Salmonella Typhimurium and Clostridium difficile in anaerobic batch culture systems: Is it due to synergies in probiotic mixtures or the specificity of single strains? *Anaerobe* 24, 60–65 (2013). 10.1016/j.anaerobe.2013.09.011 24091275

[39] Lake, F. B., van Overbeek, L. S., Baars, J. J. P., Abee, T. & den Besten, H. M. W. Growth performance of Listeria monocytogenes and background microbiota from mushroom processing environments. *Int J. Food Microbiol.* **395**, 110183 (2023). 37001480 10.1016/j.ijfoodmicro.2023.110183

[40] Dygico, L. K. et al. Lactococcus Lactis subsp. Lactis as a natural anti-listerial agent in the mushroom industry. *Food Microbiol.* **82**, 30–35 (2019). 31027787 10.1016/j.fm.2019.01.015

[41] Martín, I., Rodríguez, A., Delgado, J. & Córdoba, J. J. Strategies for Biocontrol of Listeria monocytogenes Using Lactic Acid Bacteria and Their Metabolites in Ready-To-Eat Meat-and Dairy-Ripened Products. *Foods* vol. 11 Preprint at (2022). 10.3390/foods11040542 10.3390/foods11040542 PMC8871320 35206018

[42] Lake, F. B., van Overbeek, L. S., Baars, J. J. P., Abee, T. & den Besten, H. M. W. Variability in growth and biofilm formation of Listeria monocytogenes in agaricus bisporus mushroom products. *Food Res. Int.* **165**, 112488 (2023). 36869500 10.1016/j.foodres.2023.112488

[43] Burgos, C., Melian, C., Mendoza, L. M., Salva, S. & Castellano, P. Probiotic Potential of Lactic Acid Bacteria Strains Isolated from Artisanal Cheeses: Impact on Listeria monocytogenes Infection. *Fermentation* 11, (2025).

